# Supplementary material for: Utilization of non-pneumatic anti-shock garment for treating obstetric hemorrhage and associated factors among obstetric care providers in Ethiopia: A systematic review and meta-analysis
Source: PLoS One. 2023 Nov 16;18(11):e0294052. doi: 10.1371/journal.pone.0294052 (PMC10653477; doi:10.1371/journal.pone.0294052)
Supplement: S2 Table — (DOCX) [file pone.0294052.s002.docx]

**S2 Table:** Searching strategies for some databases to assess the pooled level of Non-pneumatic Anti-shock Garment (NASG) utilization for the treatment of obstetric hemorrhage and its associated factors in Ethiopia

| **Databases** | **Searching terms** | **Number of studies** | **Searching period** |
| --- | --- | --- | --- |
| PubMed/ MEDLINE | Non-pneumatic[All Fields] AND Anti-shock[All Fields] AND ("clothing"[MeSH Terms] OR "clothing"[All Fields] OR "garment"[All Fields]) AND ("associated factors"[All Fields] OR "Factors"[All Fields] OR "determinants"[All Fields]) AND ("ethiopia"[MeSH Terms] OR "ethiopia"[All Fields]) | 22 | **From 2010/01/01 to 2023/01/18** |
| Google scholar | "Non-pneumatic Anti-shock Garment" and "utilization" and ("associated factors" or "determinants") and "Ethiopia" | 13 |  |
| DOAJ | Non-pneumatic Anti-shock Garment and associated factors and Ethiopia | 4 |  |
| African Journals Online | Non-pneumatic Anti-shock Garment and associated factors | 9 |  |
| Others database |  | 3 |  |
| Total searched articles |  | 51 |  |
| Finally, fulfill the eligibility criteria for our review |  | 8 |  |
