# Supplementary material for: Utilization of non-pneumatic anti-shock garment for treating obstetric hemorrhage and associated factors among obstetric care providers in Ethiopia: A systematic review and meta-analysis
Source: PLoS One. 2023 Nov 16;18(11):e0294052. doi: 10.1371/journal.pone.0294052 (PMC10653477; doi:10.1371/journal.pone.0294052)
Supplement: S3 Table — (DOCX) [file pone.0294052.s003.docx]

**S3 Table: Quality assessment of articles using Newcastle - Ottawa quality assessment Scale (NOS): (Adapted for cross-sectional studies)**

| Studies | **Selection** | | | | **Comparability** | **Outcome** | | **Total score** | **Quality of the study** |
| --- | --- | --- | --- | --- | --- | --- | --- | --- | --- |
|  | Representativeness  (1) | Sample size  (1) | Non-respondents  (1) | Ascertainment of the exposure (risk factor)  (2) | The subjects in different outcome groups are comparable, based on the study design or analysis. Confounding factors are controlled (2) | Assessment of the outcome  (2) | Statistical test  (1) |  |  |
| Ababo et al | * |  | * | ** | * | ** | * | 8 | High quality |
| Bekele et al | * | * | * | * | * | ** | * | 8 | High quality |
| Desta et al | * | * | * | * | ** | ** | * | 9 | High quality |
| Bekele et al | * | * | * | * | * | ** | * | 8 | High quality |
| Kettema et al | * |  | * | * | * | ** | * | 7 | High quality |
| Fentahun et al | * | * | * | * | * | * | * | 7 | High quality |
| Yeshitila et al | * | * | * | * | ** | ** | * | 9 | High quality |

**Descriptions of quality measurement adapted for cross sectional study**

**Selection: (Maximum 5 stars or 5 points)**

1) Representativeness of the sample:

1. Truly representative of the average in the target population. * (all subjects or random sampling): **1 point**
2. Somewhat representative of the average in the target population. * (nonrandom sampling) : **1 point**
3. Selected group of users: **0**
4. d) No description of the sampling strategy: 0

2) Sample size:

1. Justified and satisfactory: **1 point**
2. Not justified: **0**

3) Non-respondents:

1. Comparability between respondents and non-respondents characteristics is established, and the response rate is satisfactory: **1 point**
2. The response rate is unsatisfactory, or the comparability between respondents and non-respondents is unsatisfactory: **0**
3. No description of the response rate or the characteristics of the responders and the non-responders: **0**

4) Ascertainment of the exposure (risk factor):

1. Validated measurement tool : **(2points)**
2. Non-validated measurement tool, but the tool is available or described: **(1 point)**
3. No description of the measurement tool. **0**

**Comparability: (Maximum 2 stars or 2 points)**

1) The subjects in different outcome groups are comparable, based on the study design or analysis. Confounding factors are controlled.

1. The study controls for the most important factor (select one): 1 point
2. The study control for any additional factor: 1 point

**Outcome: (Maximum 3 points)**

1) Assessment of the outcome:

1. Independent blind assessment: **2 points**
2. Record linkage: **2 points**
3. Self-report: **1 point**
4. No description: **0**

2) Statistical test:

1. The statistical test used to analyse the data is clearly described and appropriate, and the measurement of the association is presented, including confidence intervals and the probability level (p value): **1 point**
2. The statistical test is not appropriate, not described or incomplete. **0**

**Decisions of on the quality of the studies were based on the sum or total score:**

- **High quality studies: 7-10 points**
- **Low quality studies: 0-6 points**

**S3 Table: Quality of assessment of articles using Newcastle - Ottawa quality assessment Scale (NOS) Adapted for cohort studies**

| Studies | **Selection** | | | | **Comparability** | **Outcome** | | | **Total score** | **Quality of the study** |
| --- | --- | --- | --- | --- | --- | --- | --- | --- | --- | --- |
|  | Representativeness of the exposed cohort  (2) | Selection of the non- exposed cohort  (1) | Ascertainment of exposure  (2) | outcome of interest was not present at start of study (1) | Comparability of cohorts on the basis of the design or analysis (2) | Assessment of outcome  (2) | Was follow-up long enough for outcomes to occur(1) | Adequacy of follow up of cohorts  (2) |  |  |
| Kassie et al | ** | * | ** | * | ** | ** | * | ** | 13 | High quality |
|  |  |  |  |  |  |  |  |  |  |  |
