# Supplementary material for: Utilization of non-pneumatic anti-shock garment for treating obstetric hemorrhage and associated factors among obstetric care providers in Ethiopia: A systematic review and meta-analysis
Source: PLoS One. 2023 Nov 16;18(11):e0294052. doi: 10.1371/journal.pone.0294052 (PMC10653477; doi:10.1371/journal.pone.0294052)
Supplement: Attachment — Submitted filename: Comments for Author.docx [file pone.0294052.s004.docx]

Comments for Author

1. Why you limited the date from January 1, 2010 and January 1, 2023?

Since after you have searched with different Google engine, only 8 studies were found

For to do subgroup analysis, it is recommended more than 12 studies

2. Separately write that you found final 64 studies in each data base (PubMed, Google Scholar, the African Journal of Online, ScienceDirect and the Cochrane Library)

3. You did not do sub group analysis by region, why?

4. No Meta regression in your systematic review?

5. What is your reference point sample size >300 and <- 300 in your subgroup analysis.
