# Supplementary material for: Utilization of non-pneumatic anti-shock garment for treating obstetric hemorrhage and associated factors among obstetric care providers in Ethiopia: A systematic review and meta-analysis
Source: PLoS One. 2023 Nov 16;18(11):e0294052. doi: 10.1371/journal.pone.0294052 (PMC10653477; doi:10.1371/journal.pone.0294052)
Supplement: Attachment — Submitted filename: Response to Reviewers.docx [file pone.0294052.s005.docx]

**Authors’ Point-by-Point Response to the reviewers’ and editors’ Reports**

**Title:** **Utilization of Non-pneumatic Anti-shock Garment for Treating Obstetric Hemorrhage and Associated Factors among Obstetric Care Providers in Ethiopia: A Systematic review and Meta-analysis**

**Authors:**

Dagne Addisu: [addisudagne7@gmail.com](mailto:addisudagne7@gmail.com)

Natnael Atnafu Gebeyehu: jossyatnafu2020@gmail.com

Yismaw Yimam Belachew: [yismawyimam17g@gmail.com](mailto:yismawyimam17g@gmail.com)

Maru Mekie: maru.mekie1@gmail.com

Journal: PLOS ONE

Article type: Systematic review and meta-analysis

Submission ID: PONE-D-23-02020

**Point by point response to academic editor’s comment**

Dear Editor,

We are grateful for your consideration of this manuscript, and we also very much appreciate your suggestions, which have been very helpful in improving the quality and impact of the manuscript. We have addressed all the concerns in a point by point manner and have accordingly revised the manuscript. We have highlighted the response in the response letter as well as in the revised manuscript.

**Comment:** Before our final decision, please resubmit your manuscript with point-by-point responses to the editor and reviewers (your MS needs major revision, follow journal requirements). Mandatory revisions to improve technical quality of the paper entail, but not limited to, strictly follow the journal “authors’ submission guideline” to organize the manuscript.

Response: We really appreciate this important comment. We have revised the manuscript based on the submission guidelines.

**Comment**: Refine background section should not be copy-paste from the introduction section, rewrite these sections.

Response: we would like to thank the editor for this important comment. We have revised the background.

**Comment**: Language edition of the MS, ensure substantial grammatical correction, careful language edition by experts. The resubmission should comply with our journal requirement for the standard language, refrain from typos/brevity, abbreviations, duplication of ideas, and sharpen operational definitions in the MS.

Response: thank for this useful comment. We have read the manuscript exhaustively and revised all grammatical and typing errors

**Comment**: Methods section: Carefully revise methods section, for example, clarify how the systematic review and meta-analysis conducted without registering and checking PROSPERO?

Response: Thanks for the positive comment. At the time of manuscript submission, the protocol was sent to PROSPERO for registration. Currently, the protocol of this systematic review and meta-analysis was registered at the Prospero with a registration number of (PROSPERO 2023: CRD42023414043) that is available from <https://www.crd.york.ac.uk/PROSPERO>.

**Comment:** Follow our journal requirement for reviews and meta-analysis. Recheck search strategy, variable definitions, inclusion/exclusion criteria.

Response: thanks for the kind comment. We have checked the search strategies

**Comment**: Data analysis needs to be refined by considering analysis by region, conducting meta regression to investigate the source of heterogeneities.

Response: we would to thank the academic editor for these important suggestions. We have done subgroup analysis by using region and meta-regression as per your recommendation

**Comment**: Results section: Substantially reinvestigate and sharpen it as per the reviewers’ comment.

Response: we have revised the result section as per the reviewers’ comment.

**Comment**: Discussion section is not sharp and not strong, carefully revises it based on your data/findings.

Response: We thank academic editor very much for this useful comment. We have searched for similar studies exhaustively for discussion. However, we found a limited number of studies that were related to our study or objectives. So, we tried to revise the discussion based on the findings that we found.

**Point by point response to Reviewer 1’s comments**

We are grateful to the reviewer for detailed and constructive comments, which helped us to improve the quality of the manuscript. Your warm comment and precious time and efforts invested in improving this paper are very much appreciated. We have attached a point-by-point response to each respective comment.

**Comment: #1**. First of all, I recommend the authors to strictly follow the journal “authors’ submission guideline” to organize the manuscript. For example, give continuous line number to specifically locate the comments. The figures must be submitted as a separate file in tiff format. In addition, mentioning the author’s title (Mr, Dr…) in the author list is not advisable; you can fill such information in the system.

Response: Thank you very much for your precious time and efforts invested in improving this paper. Your insightful advice is very much appreciated. We have revised the manuscript as per your suggestion

**Comment #2**: Better to avoid the abbreviations from the abstract section; moreover, you mistakenly abbreviate NASG as NSAG in the 6th line of the background in the abstract section.

Response: We really appreciate this important comment. We have mode correction on abbreviations as per your suggestion. The journal guidelines stated that the word limit in the abstract section should not exceed 300 words. Because of this, we did not avoid abbreviations completely in the abstract section. If we write the abbreviations in full, the number of words will be higher than that of the journal requirement. As a result, we tried our best to minimize the abbreviation in the abstract section.

Abstract:

**Comment**: It lacks some key points. First, the background section must not be directly copied from the introduction section; rather some paraphrasing and synthesis are mandatory. Second, the method must address the total number of journal articles retrieved from the listed databases before screening for eligibility; by which software you analyze the data and so forth. Third, in the result section you have to mention how many obstetric care providers are involved in the studies included. Finally, the conclusion is almost a copy of the result section and needs further revision. In general, as the abstract is the stand-alone summary of the whole work you have to rewrite it by incorporating the above-mentioned points and other pertinent information.

Response: We thank the reviewer very much for this useful comment. We have revised the abstract section as per your recommendations.

Methods

**Comment #1**: How did you conduct this systematic review and meta-analysis without registering it in PROSPERO? How do you know the need of review unless you checked it in PROSPERO? Moreover, unless you register the title how do you stop further duplication by other authors before this paper is published? It is also unethical to conduct systematic review and meta-analysis in such a way.

Response: Thanks for the positive comment. At the time of manuscript submission, the protocol was sent to PROSPERO for registration. Currently, the protocol of this systematic review and meta-analysis was registered at the Prospero with a registration number of (PROSPERO 2023: CRD42023414043) that is available from <https://www.crd.york.ac.uk/PROSPERO>.

**Comment #2:** Under the “searching strategies and source of information” subheading; there is duplication of ideas that needs further proof reading. E.g. The searching strategies for google scholar was written two times.

Response: Thanks for the kind comment. It was a typing error. We have removed redundant terms and sentences.

**Comment #3**: The attempts made to access gray literature were not well expressed or poor. Even all the studies included in the analysis are published. How do you see it?

Response: Response: We would like to thank the reviewer for these important suggestions. We systematically searched grey literature or unpublished studies from Ethiopian universities' institutional repositories, mainly in Gondar, Mekelle, Bahir Dar, Jimma, Addis Abeba, and Harameya University, and we found three grey literature articles related to NASG. Regarding publication status, this meta-analysis included both published and unpublished studies. Among the included studies, seven were published in different journals, whereas one was grey literature, which was retrieved from Ethiopian universities' institutional repositories. You can check the characteristics of the included studies in Table 1.

**Comment #4:** Measurement of KA: modify the operational definition; what if the participants exactly score 50% for K; under which group you categorize them. Furthermore, did all the studies included in this review measure knowledge and attitude by the same items and operationalize them in the same way?

Response: Thanks for the kind comment. Among the included strategies, only five studies identified knowledge and attitude as the associated factors for NASG utilization. Those studies operationalized knowledge and attitude in a similar way, and those who scored more than or equal to 50% on the provided knowledge questions were classified as having good knowledge and a positive attitude.

Result

**Comment #1**: There is high heterogeneity among studies even after the subgroup analysis. This may be due to methodological or statistical problems in the primary studies. Hence, it would be better to further identify the source of the problem and come up with an acceptable value. You actually mention as a limitation, however the problem is serious enough and reanalysis must be done after further evaluation of the primary studies and excluding those of poor quality.

Response: Thanks for the positive comment. Generally, it is recommended to identify the possible source of heterogeneity during meta-analysis by using different mechanisms. But the possible source of heterogeneity may not always be found for various reasons, such as variation between studies in the characteristics of their populations, variation in outcome measured, the difference in study design, variation in methodological quality, statistical heterogeneity (the variation of effects between studies), etc. We have seen several meta-analysis studies with high heterogeneity published in high-quality journals like PLOS One, BMJ Global Health, and Cochrane Libraries. This evidence shows that we cannot always find the possible source of heterogeneity.

In the case of heterogeneity, it is recommended to deal with heterogeneity by doing random effect main meta-analysis, subgroup analysis, sensitivity analysis, and met-regression. So, we have tried our best to identify the possible source of heterogeneity by performing the above recommendation. However, the possible source of heterogeneity was not identified. As a result, we have mentioned this high heterogeneity as a limitation of this meta-analysis.

**Comment #2:** In general, I appreciate the attempts made by the authors. However, the results in this paper need further investigations; some of the measures of effects reported are not trustworthy. For example, Figure 9. “Having two or more NASG within the health facility increases the odds of NASG use for PPH treatment by 10.59-fold (OR = 10.59, 95%CI = 6.59, 17.01)”. When you look at each primary study, there is too wide confidence interval that may be due to small sample size or wrong analysis. Hence, it would be better to check the quality of the primary studies again, particularly the analysis.

Response: We really appreciate this important comment. We re-assessed the quality of the study, and a smaller sample size might be a possible cause of a wider confidence interval. Regarding the quality of the studies, the overall quality scores for these studies based on the modified Newcastle-Ottawa quality assessment tool were ≥7. So, quality of the paper was not the problem and we included the studies in the meta-analysis.

**Point by point response to Reviewer 2’s comments**

**Comment:** It is good to see such good research, the way use statistical methods, flow of ideas. It is the manuscript technically sound.

Response: We would like to acknowledge the reviewer for detailed and constructive comments, which helped us to improve the quality of the manuscript. Your warm comment and precious time and efforts invested in improving this paper are very much appreciated.

**Comment**: Why you not did sub group analysis by region? And you did not do meta regression to investigate the source of heterogeneities.

Response: we would like to thank the reviewer for these important suggestions. We have done subgroup analysis by using region and meta-regression as per your recommendation

**Point by point response to Reviewer 3’s comments**

**Comment**: I am grateful for your efforts since I find your study to be very fascinating and to cover the underrepresented populations, especially in emerging nations like Ethiopia. After mentioning this, the authors should revise the suggestions that follow

Response: We would like to acknowledge the reviewer for detailed and constructive comments, which helped us to improve the quality of the manuscript. Your warm comment and precious time and efforts invested in improving this paper are very much appreciated. Below is our point-by-point response to each respective comment

**Comment**: Abstract part, Include the software used to analysis the data

Response: thanks for the positive comment. We have included the software as per your suggestion

**Comment**: Introduction: Generally it lacks coherence

- Please be concentrated on your study population and objectives throughout the manuscript
- Write the statement of the problem with the following structure
- In the first paragraph the definition and importance of your outcome variable
- Paragraph 2 show the magnitude or severity of the problem from global to local
- Paragraph 2: consequence of the problem
- Paragraph 3: Common determinants of the utilization of Anti-Shock Garment
- Paragraph 4: efforts made to increase the utilization of Anti-Shock Garment
- Paragraph 5: show the gap clearly

Response: Thank you very much for this helpful comment. We have revised the introduction as per your suggestion

Method part

**Comment**: Avoid redundancy “whereas the searching strategy for google scholar was: "Non-pneumatic Anti-shock Garment" and "utilization" and ("associated factors" or "determinants") and "Ethiopia"

Response: Thanks for the kind comment. It was a typing error. We have removed redundant terms and sentences.

**Comment**: Avoid numbering from the inclusion criteria (write in sentence case)

Response: Thanks for the positive suggestions. We have revised the inclusion criteria as per your suggestion.

Result part

**Comment**: In the first paragraph the number in text and in fig 1 is inconsistence 64 vs 61

Response: we would like to thanks the reviewer for this important comment. It was typing error. We have revised this issue. You can check supplementary file (S2 table), figure 1 and the paragraph.

**Comment**: In the inclusion criteria you say “published between January 1, 2010 and January 1, 2023,” but in the result part “ All studies were published between 2020 and 2023 and have a low risk of bias”

Response: Thanks for the kind comment. It was a typing error. We have revised this issue. The period between ‘’January 1, 2010 to January 18, 2023" was the search period. We have searched studies that were conducted or published between ’January 1, 2010 to January 18, 2023. We have limited the search period for each database between January 1, 2010, and January 18, 2023. Unfortunately all retrieved studies that fulfilled the inclusion criteria were conducted between January 1, 2016 and April 30, 2021. So, the period between January 1, 2010 and January 1, 2023 were the search period, whereas the period between 2020 and 2023 indicates the publication period for those included studies (except for one study, all included studies were published between 2020 and 2023).

**Comment:** In subgroup analysis use another variable for example region to solve heterogeneity in steady of study design

Response: we would to thank the reviewer for this important suggestion. We have done subgroup analysis by using region as per your recommendation.

Discussion part

**Comment:** Your discussion is not strong and persuasive. So, you are expected to search exhaustively and waste your time on it. You are also expected to discuss on the implications of the findings in context of existing research.

Response: We thank the reviewer very much for this useful comment. We have searched for similar studies exhaustively for discussion. However, we found a limited number of studies that were related to our study or objectives. So, we tried to revise the discussion based on the findings that we found.

**Comment**: The paper also needs extensive grammatical correction and rephrasing

Response: Again, we thank the reviewer very much for this useful comment. We have read the manuscript exhaustively and revised all grammatical and typing errors.
