# Supplementary material for: Utilization of non-pneumatic anti-shock garment for treating obstetric hemorrhage and associated factors among obstetric care providers in Ethiopia: A systematic review and meta-analysis
Source: PLoS One. 2023 Nov 16;18(11):e0294052. doi: 10.1371/journal.pone.0294052 (PMC10653477; doi:10.1371/journal.pone.0294052)
Supplement: Attachment — Submitted filename: Response to Reviewers.docx [file pone.0294052.s006.docx]

**Authors’ Point-by-Point Response to the reviewers’ and editors’ Reports**

**Title:** **Utilization of Non-pneumatic Anti-shock Garment for Treating Obstetric Hemorrhage and Associated Factors among Obstetric Care Providers in Ethiopia: A Systematic review and Meta-analysis**

**Authors:**

Dagne Addisu: [addisudagne7@gmail.com](mailto:addisudagne7@gmail.com)

Natnael Atnafu Gebeyehu: jossyatnafu2020@gmail.com

Yismaw Yimam Belachew: [yismawyimam17g@gmail.com](mailto:yismawyimam17g@gmail.com)

Maru Mekie: maru.mekie1@gmail.com

Journal: PLOS ONE

Article type: Systematic review and meta-analysis

Submission ID: PONE-D-23-02020R1

**Point by point response to academic editor’s comment**

Dear Editor,

We are grateful for your consideration of this manuscript, and we also very much appreciate your suggestions, which have been very helpful in improving the quality and impact of the manuscript. We have addressed all the concerns in a point by point manner and have accordingly revised the manuscript.

**Comment:** Please review your reference list to ensure that it is complete and correct. If you have cited papers that have been retracted, please include the rationale for doing so in the manuscript text, or remove these references and replace them with relevant current references. Any changes to the reference list should be mentioned in the rebuttal letter that accompanies your revised manuscript. If you need to cite a retracted article, indicate the article’s retracted status in the References list and also include a citation and full reference for the retraction notice.

Response: We really appreciate this important comment. We have revised the full reference based on the submission guidelines

Comments: The paper still needs minor refinement, including proof reading. Editorial staff will follow all the requirements before processing the paper.

Response: thank for this useful comment. We have read the manuscript exhaustively and revised all grammatical and typing errors

**Point by point response to Reviewer 1’s comments**

**Comment: #1**. Almost all of the concerns raised in the previous review were well addressed. However, the manuscript still needs further proof reading. For example, look at the abbreviation in line 34 "NSAG". Furthermore, the justification given for high heterogeneity, which is "We have seen several meta-analysis studies with high heterogeneity published in high-quality journals like PLOS One, BMJ Global Health, and Cochrane Libraries". Better to give scientific justification with reference. Being published in high ranking journal does not always show the quality of the paper

Response: We really appreciate your warm comment and precious time and efforts invested in improving this paper are very much appreciated. We have understood your concern and we have revised the manuscript as per your suggestions.

**Point by point response to Reviewer 2’s comments**

Reviewer #2: The authors have made significant contributions to the subject, pointing out important gaps that need to be filled by future studies. I recommend the acceptance of this publication research manuscript for publication in this journal.

Response: We would like to acknowledge the reviewer for the kind comments. Your warm comment and precious time and efforts invested in improving this paper are very much appreciated.
